# Infantile Colic-Related Symptoms and Maternal Feeding Attitudes in Infants Aged 7–12 Months: Associations with Sensory Processing Characteristics in a Cross-Sectional Study

**DOI:** 10.3390/children13101437

**Published:** 2026-10-19

**Authors:** Yasin Tekeci, Enes Tuğrul, Melike Selin İkinci Kaya, Berkan Torpil

**Affiliations:** 1 Department of Occupational Therapy, Faculty of Gülhane Health Sciences, University of Health Sciences Türkiye, Ankara 06018, Türkiye; yasin.tekeci@sbu.edu.tr (Y.T.); berkan.torpil@sbu.edu.tr (B.T.); 2 Private Health Professional Service Unit, Antalya 07059, Türkiye; eneshcttp@gmail.com; 3 Department of Occupational Therapy, Faculty of Health Sciences, Atlas University, Istanbul 34408, Türkiye

**Keywords:** infantile colic, sensory processing, Infant/Toddler Sensory Profile-2, infant feeding attitudes, late infancy, occupational therapy

## Abstract

**Highlights:**

**What are the main findings?**

- In infants aged 7–12 months with a documented history of colic, sensory processing characteristics, particularly General Processing, were strongly associated with current colic-related symptom scores.
- Higher colic-related symptom scores and higher Low Registration scores were independently associated with less breastfeeding-positive maternal attitudes.

**What are the implications of the main findings?**

- Sensory processing may be a useful additional consideration when evaluating older infants with a history of colic who continue to show regulatory or feeding-related concerns.
- Longitudinal studies with non-colic comparison groups are needed to determine whether these associations reflect persistent developmental differences.

**Abstract:**

**Background/Objectives:** Infantile colic is primarily recognized as an early-infancy condition, yet early regulatory difficulties may be associated with sensory processing and feeding-related characteristics later in infancy. This study examined associations among colic-related symptom scores, sensory processing characteristics, and maternal attitudes toward breastfeeding versus formula feeding in infants aged 7–12 months with a documented history of infantile colic. **Methods:** This cross-sectional correlational study included 100 infants aged 7–12 months with a clinically documented colic diagnosis in the first 4 months of life meeting Wessel’s criteria. The Infant/Toddler Sensory Profile-2 (ITSP-2), Infant Colic Scale (ICS), and Iowa Infant Feeding Attitude Scale (IIFAS) were administered. Data were analyzed using Pearson correlations and two multiple linear regression models. **Results:** IIFAS scores were inversely correlated with ICS scores (r = −0.708, *p* < 0.001). ICS scores correlated most strongly with General Processing (r = 0.804), Low Registration (r = 0.749), Sensitivity (r = 0.737), and Seeking (r = 0.719; all *p* < 0.001). In regression analyses, ICS scores were independently associated with IIFAS score (β = −0.291), General Processing (β = 0.567), Sensation Avoiding (β = −0.146), and Auditory Processing (β = −0.125) (R^2^ = 0.728, adjusted R^2^ = 0.716), while IIFAS scores were independently associated with ICS score (β = −0.427) and Low Registration (β = −0.376) (R^2^ = 0.564, adjusted R^2^ = 0.555; all *p* < 0.05). **Conclusions:** In infants with a history of colic, sensory processing characteristics were associated with current colic-related symptom scores and maternal feeding attitudes and may warrant consideration in clinical evaluation. Longitudinal studies are needed to clarify the temporal and causal nature of these associations.

## 1. Introduction

Infantile colic is a common early-infancy condition characterized by recurrent episodes of prolonged, inconsolable crying in otherwise healthy infants. The classic Wessel “rule of threes” defines colic as crying for more than 3 h per day, on at least 3 days per week, for more than 3 weeks [1,2,3]. Although gastrointestinal mechanisms have historically dominated explanations of colic, only a minority of affected infants have an identifiable organic disorder, and contemporary models increasingly consider regulatory, neurodevelopmental, circadian, and sensory mechanisms [4,5,6].

Self-regulation during infancy depends on the infant’s capacity to modulate physiological arousal, sensory input, behavioral state, and interaction with caregivers [7,8]. Sensory processing—the detection, organization, and modulation of sensory information—contributes to this regulatory capacity [9]. Differences in neurological thresholds and behavioral response strategies may therefore influence how infants respond to ordinary auditory, visual, tactile, movement, and oral input [9,10]. Previous work has linked early regulatory difficulties and excessive crying with later behavioral, sensory, sleeping, and feeding difficulties, suggesting that the developmental correlates of early colic may extend beyond the period in which crying is most prominent [10,11,12,13,14,15,16]. More recently, Zorlular et al. demonstrated that regulatory and sensory difficulties associated with a history of infantile colic persist at the cusp of toddlerhood, accompanied by altered sleep and motor outcomes [17].

Feeding is a particularly relevant context in late infancy because it integrates oral sensory input, touch, movement, caregiver responsiveness, and emerging complementary-feeding routines. Parent–infant interaction is transactional: infant characteristics may shape caregiver responses, while caregiver behavior may in turn influence infant regulation [18,19,20,21,22]. Maternal attitudes toward breastfeeding and formula feeding may therefore be meaningfully related to the infant’s regulatory and sensory characteristics, although such associations should not be interpreted as evidence of directionality in cross-sectional data [18,22,23].

The 7–12-month period is also developmentally important because complementary feeding becomes established and infants encounter increasingly varied textures, tastes, and oral-motor demands [24,25]. In contrast, most infantile colic research has focused on the first 3–4 months of life [26]. Evidence regarding sensory processing characteristics and feeding-related maternal attitudes among older infants with a documented history of colic remains limited.

Accordingly, this study aimed to examine the relationships among colic-related symptom scores, maternal attitudes toward breastfeeding versus formula feeding, and sensory processing characteristics in infants aged 7–12 months with a clinically documented history of infantile colic. A secondary aim was to identify sensory processing characteristics independently associated with ICS and IIFAS scores after multivariable analysis. Given the cross-sectional design, the analyses were intended to identify associations rather than causal or longitudinal effects.

## 2. Materials and Methods

### 2.1. Study Design and Ethical Approval

This cross-sectional correlational study was conducted after approval from the Local Non-Interventional Clinical Research Ethics Committee (Decision No. May 2025-07). The study was conducted in accordance with the Declaration of Helsinki, and written informed consent was obtained from the legal guardians of all participating infants. Reporting was structured in accordance with the STROBE principles for observational studies.

### 2.2. Sample Size

An a priori sample-size calculation was performed using G*Power version 3.1.9.7 (Heinrich-Heine-Universität Düsseldorf, Germany) for multiple linear regression. Assuming a medium effect size (f^2^ = 0.15), α = 0.05, and statistical power of 80%, the minimum required sample was 70 participants. To increase precision for the planned multivariable analyses, 100 infants were included.

### 2.3. Setting and Participant Recruitment

This cross-sectional study was conducted between October 2025 and December 2025 in Antalya, Türkiye. Participants were outpatient infants who had received a formal clinical diagnosis of infantile colic during the first 4 months of life by three independent specialist pediatricians practicing in outpatient clinics and were subsequently referred to a private outpatient Occupational Therapy Clinic for developmental follow-up.

To reduce reliance on retrospective parental recall, the previous colic diagnosis was checked against available pediatric clinical records documenting the criteria used at the time of diagnosis. Records were reviewed for a history consistent with modified Wessel’s criteria.

Inclusion criteria were: (1) age 7–12 months (≥181 days); (2) a physician-documented diagnosis of infantile colic during the first 4 months of life, with clinical records indicating that Wessel’s criteria had been met; and (3) a feeding regimen consisting of continued breastfeeding supplemented with introduced complementary solid foods (all infants had an initial history of predominant/exclusive breastfeeding in the first 6 months and were currently receiving breast milk alongside complementary feeding, ensuring nutritional homogeneity); and (4) the biological mother available as the primary caregiver to complete assessments.

**Figure 1:**
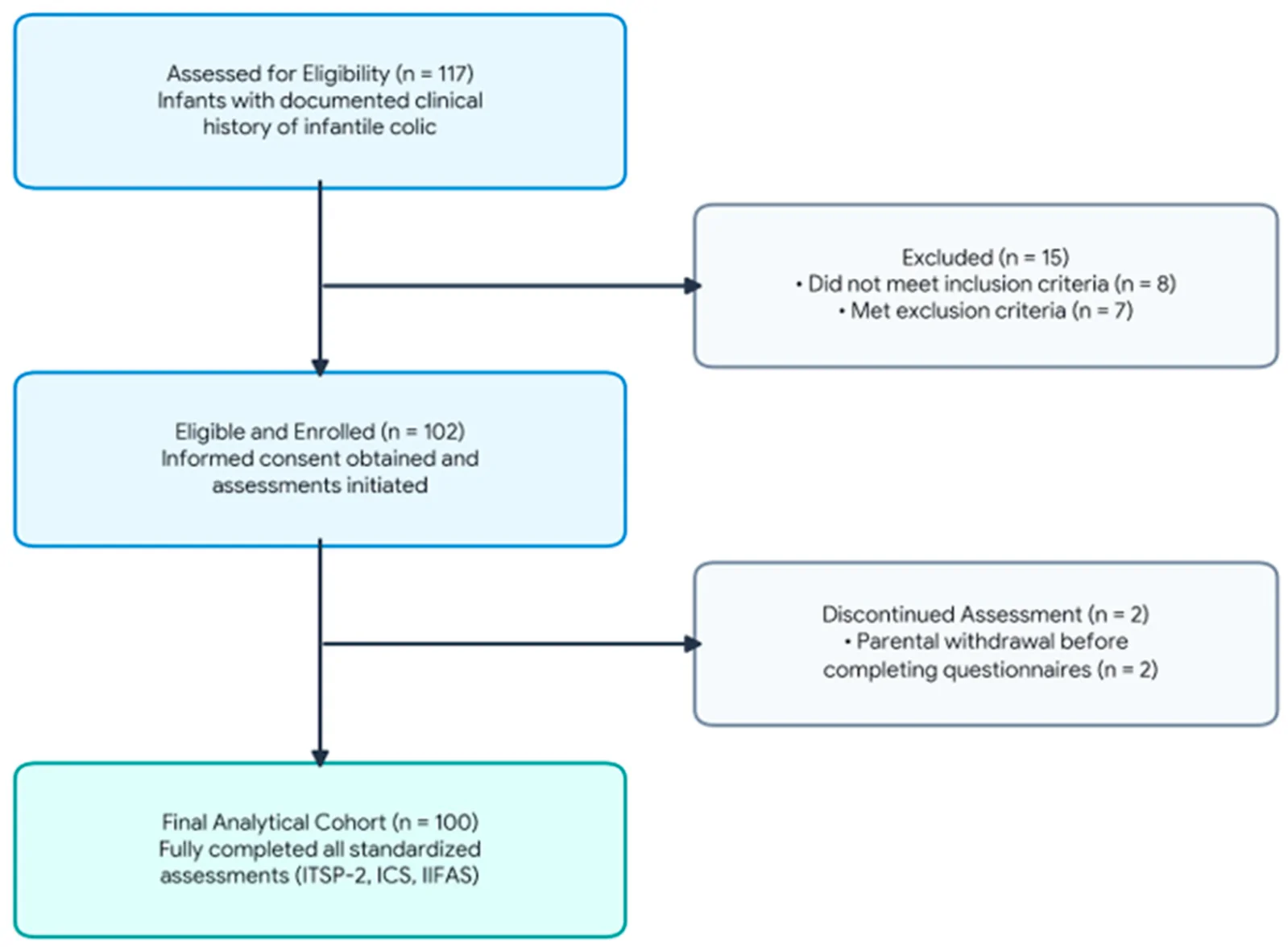
STROBE Flow Diagram of Participant Selection and Recruitment.

Infants were excluded if they had a major congenital anomaly; a diagnosed neurological, orthopedic, or genetic disorder; clinically significant visual or hearing impairment; gestational age <35 weeks or severe complications related to prematurity; pathological jaundice requiring intensive phototherapy or exchange transfusion; or any history of neonatal intensive care unit admission. A total of 117 infants with a documented history of infantile colic were assessed for eligibility. Fifteen were excluded (8 did not meet the inclusion criteria and 7 met the exclusion criteria). Of the 102 enrolled infants, 2 were withdrawn by their parents before completing the questionnaires, yielding a final sample of 100 infants who completed all assessments (Figure 1).

### 2.4. Measures

All assessment sessions were conducted in a quiet, child-friendly evaluation room at the occupational therapy clinic during scheduled outpatient appointments. To ensure methodological consistency and high data quality, all standardized instruments (ITSP-2, ICS, and IIFAS) and the sociodemographic and clinical information form were administered face-to-face through structured clinical interviews conducted directly by a licensed professional occupational therapist with the infant’s biological mother as the sole respondent. The evaluating therapist guided each mother, clarified instructions neutrally, and verified completeness to prevent missing data.

#### 2.4.1. Sociodemographic and Clinical Information Form

A structured form was used to record infant age, gestational age at birth, mode of delivery, postnatal hospital stays, nighttime sleep duration, number of nighttime awakenings, maternal age, and birth order. Infant feeding status was confirmed via direct maternal structured interview, verifying that all infants were currently receiving breast milk alongside introduced complementary solid foods. Parental educational attainment was not recorded in the clinical intake protocol.

#### 2.4.2. Infant/Toddler Sensory Profile-2

The Infant/Toddler Sensory Profile-2 (ITSP-2) was used to characterize sensory processing [27]. The caregiver-completed instrument is based on Dunn’s model of sensory processing and assesses the interaction between neurological thresholds and behavioral response strategies. For the age range examined in this study, the profile includes the sensory quadrants Low Registration, Sensation Seeking, Sensory Sensitivity, and Sensation Avoiding, together with General, Auditory, Visual, Touch, Movement, Oral, and Behavioral processing sections. Higher or lower raw scores are interpreted using age-referenced normative classification categories: Much Less Than Others, Less Than Others, Just Like the Majority of Others, More Than Others, and Much More Than Others. The validated Turkish version was used [28]. Importantly, General Processing is an ITSP-2 subscale and was not treated as a total sensory-processing score.

#### 2.4.3. Infant Colic Scale

The Infant Colic Scale (ICS), developed by Cirgin Ellett et al. [29] and adapted into Turkish by Çetinkaya and Başbakkal [30], contains 19 items rated on a 6-point Likert scale. The instrument includes domains related to cow’s milk/soy protein intolerance, immature gastrointestinal function, immature central nervous system function, difficult infant temperament, and parent–infant interaction. Higher scores indicate a greater burden of colic-related characteristics. Because the present sample was 7–12 months old and colic had been diagnosed earlier in infancy, ICS scores were interpreted conservatively as current colic-related symptom scores rather than as evidence of an active diagnostic state of infantile colic.

#### 2.4.4. Iowa Infant Feeding Attitude Scale

The Iowa Infant Feeding Attitude Scale (IIFAS) was developed by De La Mora et al. [23] to assess maternal attitudes toward breastfeeding and formula feeding. The 17-item instrument uses a 5-point Likert response format. Items favoring formula feeding are reverse-scored, producing a total score from 17 to 85; higher scores reflect a more positive attitude toward breastfeeding, whereas lower scores reflect a more positive attitude toward formula feeding. The validated Turkish version was used [31]. Accordingly, IIFAS scores in this study were not interpreted as a direct measure of feeding quality, responsive feeding, or mother–infant interaction.

### 2.5. Control of Potential Sources of Bias

Several methodological steps were undertaken to address potential sources of bias:

1. Selection and Recall Bias: Early colic diagnoses were checked against pediatric clinical records, reducing reliance on parental recall of diagnosis. Restricting enrollment to infants receiving breast milk alongside complementary foods reduced variation in broad feeding modality within the sample, but did not remove residual confounding or selection bias. Exclusion criteria restricted several major neonatal and neurodevelopmental conditions.
2. Information and Measurement Bias: All assessments were completed in face-to-face interviews with biological mothers and facilitated by the same licensed occupational therapist. This standardized administration allowed clarification of instructions, but maternal report, interviewer effects, and common-method bias remain possible. The study team checked questionnaires for completeness.
3. Confounding and Collinearity: VIF and tolerance statistics were examined to assess collinearity among predictors in the retained regression models. These diagnostics do not control for confounding. Unmeasured factors, including parental education and other feeding-related characteristics, may have influenced the observed associations.

### 2.6. Statistical Analysis

All analyses were performed using JASP version 0.19.3 (JASP Team, University of Amsterdam, Amsterdam, The Netherlands). Continuous variables are presented as mean ± standard deviation (SD) and range, and categorical variables as frequency and percentage. Distributional assumptions were evaluated using skewness and kurtosis together with visual inspection of histograms and Q–Q plots. The continuous study variables showed approximately normal distributions, supporting the use of parametric analyses.

For the ITSP-2, frequencies and percentages were calculated for each age-referenced normative classification category. Pearson correlation coefficients were used to examine associations among IIFAS, ICS, and ITSP-2 scores. For descriptive interpretation, absolute r values of 0.10–0.29, 0.30–0.49, and ≥0.50 were considered weak, moderate, and strong, respectively.

Two multiple linear regression models were used to examine variables independently associated with (1) ICS scores and (2) IIFAS scores. Candidate variables were evaluated with attention to bivariate associations and collinearity because several ITSP-2 dimensions were strongly intercorrelated. Model assumptions—linearity, normally distributed residuals, homoscedasticity, independence of errors, and multicollinearity—were assessed before interpretation. Variance inflation factor and tolerance statistics were examined and did not indicate problematic multicollinearity in the retained models. Regression coefficients are reported as unstandardized B, standard error (SE), standardized β, t statistics, *p* values, and 95% confidence intervals where available. All tests were two-tailed and *p* < 0.05 was considered statistically significant. A correlation heatmap was produced as a descriptive visualization.

Generative artificial intelligence tools were used in the preparation of the figures.

## 3. Results

### 3.1. Participant Characteristics

The study included 100 infants with a mean age of 9.89 ± 1.41 months. Seventy-three percent were born by cesarean section. Mean maternal age at delivery was 28.17 ± 2.75 years, and 74% of the infants were first-born children. All participants resided in urban metropolitan areas. Additional characteristics are presented in Table 1.

### 3.2. Scale Scores and Sensory Classification

Mean ICS and IIFAS scores were 63.95 ± 5.84 and 55.50 ± 7.20, respectively. ITSP-2 raw scores are summarized in Table 2. Because ITSP-2 sections contain different numbers of items and different normative ranges, raw means should not be used to rank the relative severity of sensory-processing domains.

Normative ITSP-2 classifications showed domain-specific patterns (Table 3). Most infants were classified as just like the majority of others for Avoiding (66%), Sensitivity (60%), Registration (60%), Movement (50%), and Oral Processing (62%). In contrast, 52% were classified as Much More Than Others for Touch Processing, 48% for Behavioral Responses, and 42% for General Processing. Visual Processing showed a different pattern, with 56% classified as Less Than Others. These distributions describe departures from age-referenced normative categories and should not be interpreted as direct comparisons with a concurrently recruited control group.

### 3.3. Correlations Among Feeding Attitudes, Colic-Related Symptoms, and Sensory Processing

IIFAS scores were inversely correlated with ICS scores (r = −0.708, *p* < 0.001). IIFAS scores were also significantly associated with most sensory dimensions, with significant coefficients ranging in absolute magnitude from 0.227 to 0.696; the strongest inverse associations were observed for Registration (r = −0.696), General Processing (r = −0.680), Sensitivity (r = −0.632), Seeking (r = −0.618), Touch Processing (r = −0.613), and Behavioral Responses (r = −0.610; all *p* < 0.001). Avoiding was positively correlated with IIFAS scores (r = 0.414, *p* < 0.001).

**Figure 2:**
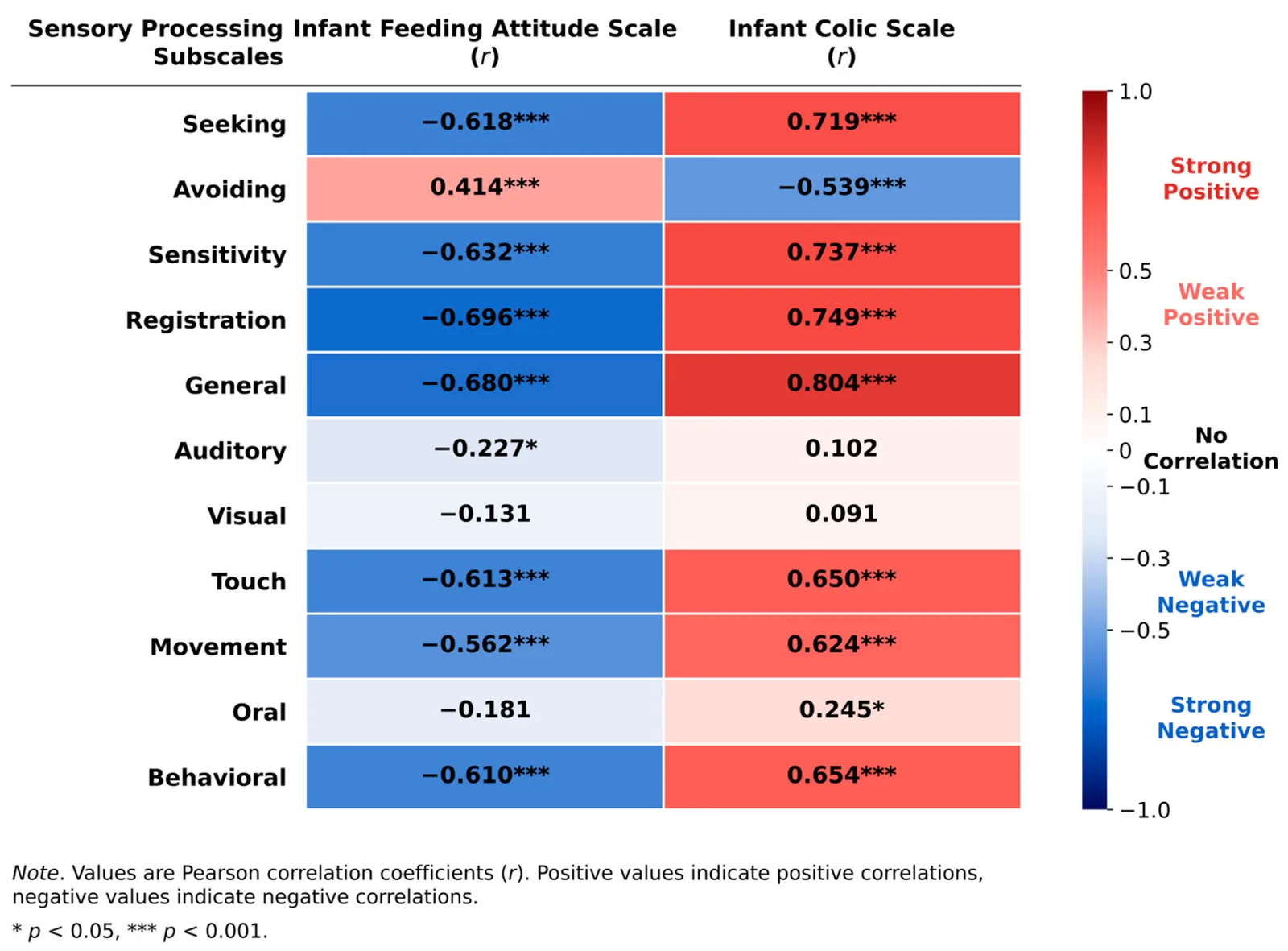
Heatmap of Pearson correlations between sensory processing dimensions and the two principal study outcomes. Color intensity represents correlation magnitude and direction. The figure is descriptive; exact coefficients and significance levels are reported in Table 4.

ICS scores showed significant associations with several sensory dimensions. Positive correlations ranged from r = 0.245 for Oral Processing to r = 0.804 for General Processing; particularly strong associations were observed for General Processing, Registration, Sensitivity, Seeking, Touch Processing, Behavioral Responses, and Movement Processing. Avoiding was inversely correlated with ICS scores (r = −0.539, *p* < 0.001), whereas Auditory and Visual Processing were not significantly correlated with ICS scores (Figure 2).

**Table 4:** Pearson correlation coefficients among study variables (*N* = 100).

Variable	1	2	3	4	5	6	7	8	9	10	11	12	13
1. Iowa Infant Feeding Attitude Scale	—												
2. Infant Colic Scale	−0.708 **	—											
3. Seeking	−0.618 **	0.719 **	—										
4. Avoiding	0.414 **	−0.539 **	−0.405 **	—									
5. Sensitivity	−0.632 **	0.737 **	0.822 **	−0.429 **	—								
6. Registration	−0.696 **	0.749 **	0.645 **	−0.454 **	0.809 **	—							
7. General Processing	−0.680 **	0.804 **	0.831 **	−0.497 **	0.911 **	0.829 **	—						
8. Auditory	−0.227 *	0.102	0.222 *	−0.071	0.231 *	0.176	0.266 **	—					
9. Visual	−0.131	0.091	0.110	0.044	0.053	0.132	0.058	−0.080	—				
10. Touch	−0.613 **	0.650 **	0.720 **	−0.215 *	0.672 **	0.610 **	0.702 **	0.150	0.140	—			
11. Movement	−0.562 **	0.624 **	0.744 **	−0.310 **	0.646 **	0.537 **	0.676 **	0.343 **	0.185	0.678 **	—		
12. Oral	−0.181	0.245 *	0.287 **	−0.094	0.231 *	0.228 *	0.209 *	0.007	0.102	0.125	0.289 **	—	
13. Behavioral	−0.610 **	0.654 **	0.692 **	−0.346 **	0.686 **	0.644 **	0.680 **	0.201 *	0.140	0.675 **	0.545 **	0.251 *	—

Values are Pearson correlation coefficients (r). * *p* < 0.05; ** *p* < 0.001. Positive coefficients indicate direct associations and negative coefficients indicate inverse associations.

### 3.4. Multiple Linear Regression Analyses

**Figure 3:**
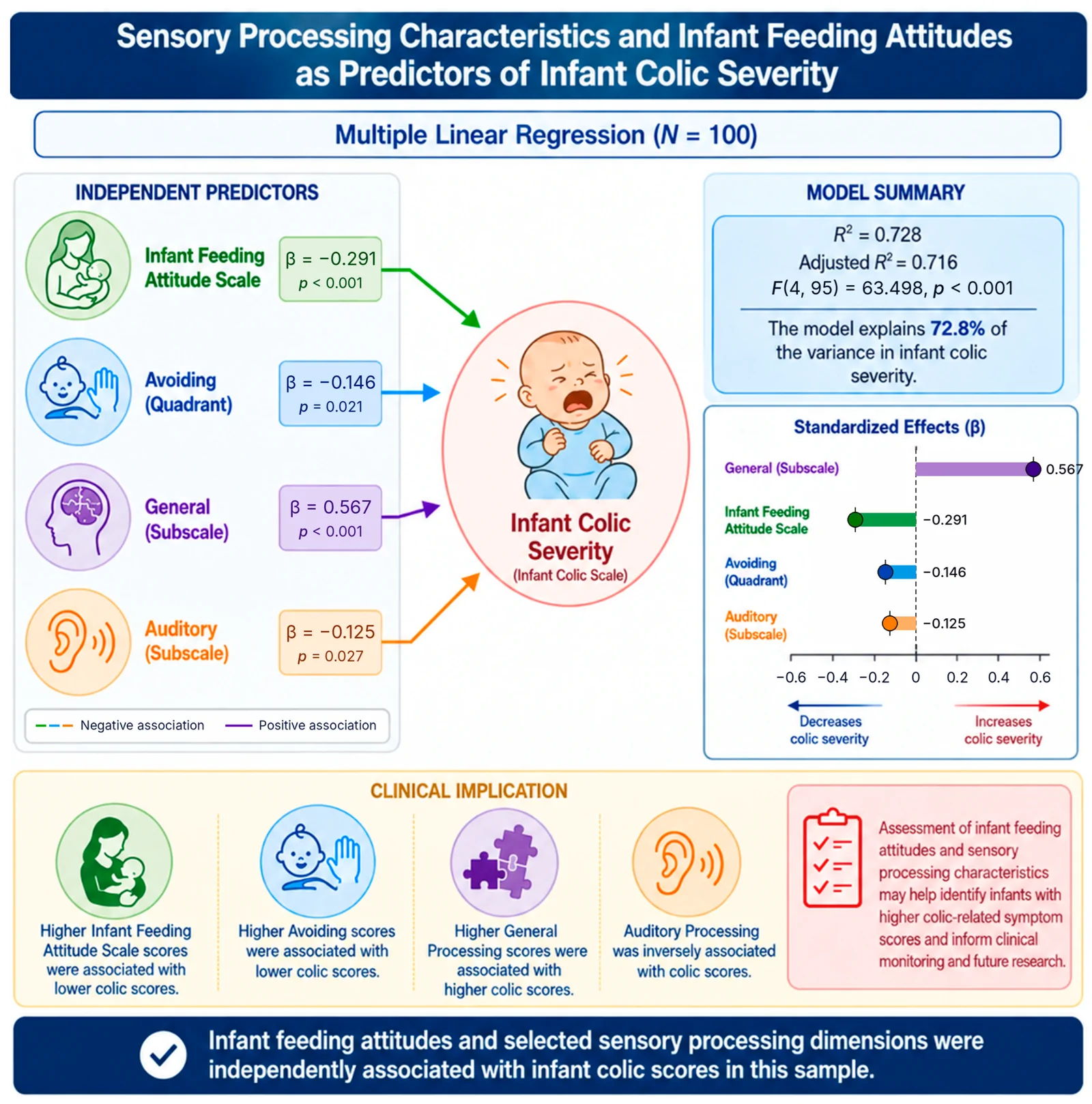
Visual summary of the multiple linear regression model for Infant Colic Scale (ICS) scores. Values represent standardized regression coefficients (β) and are interpreted as adjusted cross-sectional associations, not causal effects. Higher Iowa Infant Feeding Attitude Scale (IIFAS) scores reflect more breastfeeding-positive maternal attitudes.

The regression model for ICS scores was statistically significant, F(4, 95) = 63.498, *p* < 0.001, and explained 72.8% of the variance (R^2^ = 0.728; adjusted R^2^ = 0.716). General Processing showed the largest standardized association with ICS scores (β = 0.567, *p* < 0.001). Higher IIFAS scores were independently associated with lower ICS scores (β = −0.291, *p* < 0.001). Avoiding (β = −0.146, *p* = 0.021) and Auditory Processing (β = −0.125, *p* = 0.027) were also independently associated with ICS scores. These coefficients represent adjusted cross-sectional associations and do not establish causal effects (Figure 3, Table 5).

**Figure 4:**
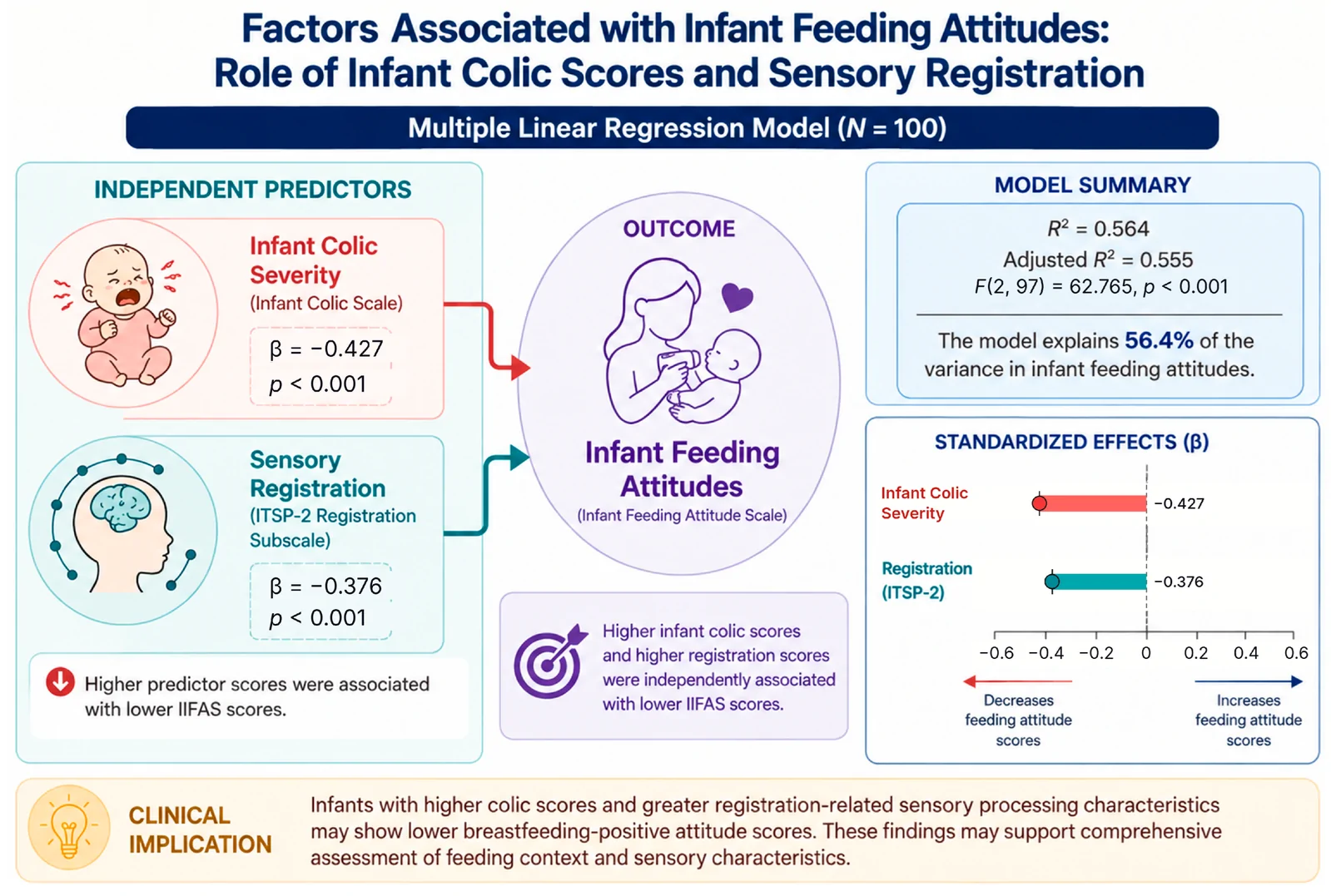
Visual summary of the multiple linear regression model for Iowa Infant Feeding Attitude Scale (IIFAS) scores. Higher IIFAS scores indicate more breastfeeding-positive attitudes; negative coefficients indicate associations with lower IIFAS scores, not poorer feeding quality or impaired mother–infant interaction. Associations are cross-sectional and do not imply causality.

The regression model for IIFAS scores was statistically significant, F(2, 97) = 62.765, *p* < 0.001, and explained 56.4% of the variance (R^2^ = 0.564; adjusted R^2^ = 0.555). Higher ICS scores were independently associated with lower IIFAS scores (β = −0.427, *p* < 0.001), and higher Registration scores were independently associated with lower IIFAS scores (β = −0.376, *p* < 0.001). Because higher IIFAS scores indicate a more positive attitude toward breastfeeding, these findings indicate that greater colic-related symptom burden and higher Low Registration scores were associated with comparatively less breastfeeding-positive maternal attitudes; they do not directly measure feeding quality or caregiver responsiveness (Table 6, Figure 4).

## 4. Discussion

This study examined sensory processing characteristics, colic-related symptom scores, and maternal feeding attitudes in 7–12-month-old infants with a clinically documented history of infantile colic. Three findings are central. First, ITSP-2 normative classifications showed marked domain-specific variation, with relatively large proportions of infants classified as Much More Than Others for Touch Processing, Behavioral Responses, and General Processing, while most infants remained within the normative majority for several other dimensions. Second, ICS scores were strongly associated with multiple sensory processing dimensions and inversely associated with IIFAS scores. Third, General Processing, Avoiding, Auditory Processing, and IIFAS scores were independently associated with ICS scores, whereas ICS and Low Registration scores were independently associated with maternal attitudes toward breastfeeding versus formula feeding.

The sensory classification findings should be interpreted relative to ITSP-2 normative categories rather than as direct evidence that infants with a history of colic differ from a concurrently recruited non-colic control group. Nevertheless, the distribution suggests that sensory characteristics may remain clinically relevant during late infancy in at least a subset of infants with an earlier history of colic. This interpretation is consistent with longitudinal and follow-up literature linking early crying and regulatory difficulties with later sensory, behavioral, sleep, and feeding outcomes [10,11,12,13,14,15,16]. Because the present study did not measure sensory processing during the acute colic period, however, it cannot determine whether the observed characteristics represent persistence of earlier sensory differences, later-emerging features, or stable individual variation.

Previous studies have suggested that tactile responsivity and broader sensory regulation may be relevant to infants with colic or early regulatory difficulties [9,13,15]. Zorlular et al. [17] reported sensory processing findings during the transition toward toddlerhood among infants with a history of colic. In the present sample, 52% were categorized as ‘Much More Than Others’ on the Touch Processing section of the ITSP-2. This norm-referenced categorization does not in itself establish tactile defensiveness, developmental persistence, or a causal consequence of colic. General Processing and Behavioral Responses also showed relatively high proportions in the ‘Much More Than Others’ category, suggesting a heterogeneous pattern of sensory-related behaviors.

These findings may inform pediatric and occupational therapy assessment, particularly when older infants with a history of colic present with feeding or regulation concerns. Sensory processing differences could be considered when clinicians explore reported reactions to touch, routine care, or complementary-food textures; however, the present study did not directly measure food refusal, latch mechanics, facial cleansing responses, or the effectiveness of intervention strategies. Any individualized advice should therefore be based on a comprehensive clinical assessment rather than ITSP-2 category membership alone. Prospective research is needed to determine whether sensory-informed parent guidance, environmental adaptations, or graded exposure to textures improve outcomes.

Other sensory domains showed more heterogeneous patterns. Half of the infants were within the normative majority for Movement Processing, and only 18% were classified above the normative majority; thus, the data do not support a generalized claim of elevated vestibular responsivity across the sample. Similarly, 62% were within the normative majority for Oral Processing, although 38% were classified above it. These findings are relevant in the context of complementary feeding, when infants encounter increasing texture, taste, and oral-motor demands [24,25], but the present study did not directly assess oral-motor function, food refusal, or feeding performance. Visual Processing differed in the opposite direction, with 56% classified as Less Than Others. This heterogeneity reinforces the need to examine specific sensory dimensions rather than infer a uniform sensory phenotype.

The correlation pattern was substantial. ICS scores were strongly related to General Processing, Registration, Sensitivity, Seeking, Touch Processing, Behavioral Responses, and Movement Processing. These associations are compatible with neurodevelopmental models that conceptualize excessive crying and regulatory difficulties as involving interactions among sensory thresholds, arousal regulation, and caregiver–infant processes [9,17,21,32,33,34]. However, the strong intercorrelations among several ITSP-2 dimensions also indicate that these constructs overlap statistically. The multivariable results should therefore be interpreted as estimates conditional on the other variables retained in each model rather than as evidence that individual sensory systems operate independently.

In the ICS regression model, General Processing showed the largest standardized coefficient. This finding indicates that, within the fitted model, broader general sensory-processing characteristics were most strongly associated with variation in colic-related symptom scores. Avoiding and Auditory Processing had smaller inverse adjusted coefficients. The direction of these coefficients differs from some of their unadjusted relationships, a pattern that can occur when correlated sensory dimensions are modeled simultaneously. This is another reason to avoid mechanistic interpretation of individual coefficients and to emphasize the multivariable association pattern as a whole.

IIFAS scores were inversely correlated with ICS scores and with several sensory-processing dimensions. In the multivariable model, higher ICS and Low Registration scores were independently associated with lower IIFAS scores. Importantly, IIFAS is an attitudinal measure: lower scores indicate comparatively more formula-positive and less breastfeeding-positive attitudes, not poorer feeding quality or deficient maternal responsiveness [23,31]. The association may nevertheless be clinically relevant because feeding attitudes develop within a broader caregiver–infant context. Infants who are more difficult to regulate or who show reduced registration of sensory and social cues may contribute to a more challenging feeding experience, while maternal feeding preferences and expectations may also shape interaction. The cross-sectional design cannot determine the direction of these relationships.

The relationship between sensory processing and maternal feeding attitudes should be interpreted in the context of evidence concerning breastfeeding practices and postnatal support. Roșca et al. [35] examined maternal feeding practices and associated factors in a cross-sectional survey, while Constantin et al. [36] examined associations between hospital practices, early postnatal support, and breastfeeding outcomes. Although these studies provide relevant context, neither directly establishes a pathway from infant sensory registration to maternal attitudes. In our exclusively breastfeeding-plus-complementary-feeding cohort, higher Registration scores were associated with lower IIFAS scores after adjustment for ICS scores. One possible explanation is that infant sensory responsiveness and caregiver perceptions interact during feeding; however, hunger cues, latch mechanics, feeding performance, maternal stress, and perceived milk adequacy were not directly assessed. These mechanisms therefore remain hypotheses for future research rather than findings of the present study.

These findings have potential implications for pediatric assessment. In infants with a history of colic who continue to present with regulatory or feeding-related concerns during late infancy, assessment of sensory processing may provide complementary information about the infant’s behavioral responses to everyday input. The results do not establish the effectiveness of sensory-based intervention, sensory diets, environmental modification, or parent-mediated co-regulation strategies. Such interventions require direct evaluation in prospective and controlled studies before treatment recommendations can be made.

An important design feature was the restriction of the sample to infants receiving breast milk alongside complementary foods, which reduced between-participant variation in broad feeding modality. However, this restriction limits generalizability and does not control for variation in breastfeeding frequency, complementary food exposure, maternal preferences, or other unmeasured confounders. Other strengths include checking early colic diagnoses against pediatric clinical records and using a consistent, face-to-face administration procedure.

The likely directions of potential biases warrant consideration, although their magnitudes cannot be quantified from these data. Recruitment following pediatric referral to a specialized occupational therapy clinic may have favored families seeking additional developmental evaluation or infants with more noticeable concerns. This may have shifted symptom-score distributions upward relative to a general community sample; its effect on correlation or regression coefficients is uncertain and could operate in either direction. Because the same mother reported the ICS, ITSP-2, and IIFAS responses, correlated reporting tendencies could have inflated some observed associations, although the magnitude is unknown. Standardized interviewer-assisted administration may have reduced misunderstanding or missing responses, but it cannot eliminate shared-method bias or interviewer influence.

This study has several limitations. Its cross-sectional design precludes inferences about causality, temporal direction, or persistence of early sensory differences. No non-colic comparison group was enrolled; ITSP-2 normative classifications should not be read as direct case-control contrasts. The sample was recruited from a single urban outpatient occupational therapy setting and was restricted to infants receiving breast milk alongside complementary foods, limiting generalizability to community samples, exclusively formula-fed infants, preterm infants, and different settings or cultures. Parental education was not collected, and other potential confounders—including breastfeeding intensity, maternal mental health, and feeding difficulties—were not fully characterized. The same maternal respondent completed all scales, creating a risk of shared-method bias. Finally, the ICS was applied at 7–12 months, beyond the typical acute period of infantile colic; scores are therefore described as colic-related characteristics rather than evidence of active colic.

## 5. Conclusions

In 7–12-month-old infants with a documented history of infantile colic, sensory processing characteristics were closely associated with current colic-related symptom scores and maternal attitudes toward breastfeeding versus formula feeding. General Processing showed the strongest independent association with ICS scores, while Low Registration and ICS scores were independently associated with IIFAS scores. The findings support a broader developmental assessment perspective for infants with a history of colic, but they do not establish persistence, causality, or treatment effects. Longitudinal studies with non-colic comparison groups and direct feeding and developmental assessments are needed to clarify the clinical significance and developmental trajectory of these associations.

## Figures and Tables

**Table 1:** Descriptive characteristics of infants and mothers.

Variable	*n* (%) or Mean ± SD	Range
Infant age, months	9.89 ± 1.41	7–12
Mode of delivery		
Cesarean	73 (73.0)	—
Vaginal	27 (27.0)	—
Postnatal hospital stay, days	2.03 ± 1.09	1–7
Nighttime awakenings, *n*	2.38 ± 1.70	0–6
Nighttime sleep duration, h	9.81 ± 1.46	7–12
Maternal age at delivery, years	28.17 ± 2.75	23–36
Birth order		
First child	74 (74.0)	—
Second child	26 (26.0)	—

Continuous variables are presented as mean ± SD; categorical variables are presented as *n* (%).

**Table 2:** Descriptive statistics for colic-related symptoms, maternal feeding attitudes, and sensory processing measures (*n* = 100).

Variable	Mean ± SD	Range
Infant Colic Scale	63.95 ± 5.84	52–75
Iowa Infant Feeding Attitude Scale	55.50 ± 7.20	42–67
Infant/Toddler Sensory Profile-2		
Seeking	21.69 ± 6.79	11–34
Avoiding	17.23 ± 5.02	6–29
Sensitivity	20.28 ± 7.12	9–35
Registration	17.50 ± 6.03	8–29
General Processing	25.56 ± 11.05	10–44
Auditory Processing	14.76 ± 4.26	7–27
Visual Processing	10.33 ± 2.37	6–17
Touch Processing	17.89 ± 6.02	6–30
Movement Processing	14.85 ± 5.34	5–24
Oral Processing	14.87 ± 5.31	7–28
Behavioral Responses	16.84 ± 6.55	7–29

**Table 3:** Distribution across Infant/Toddler Sensory Profile-2 normative classification categories (*n* = 100).

ITSP-2 Dimension	Much Less than Others *n* (%)	Less than Others *n* (%)	Just Like the Majority *n* (%)	More than Others *n* (%)	Much More than Others *n* (%)
Seeking	34 (34.0)	29 (29.0)	34 (34.0)	3 (3.0)	0 (0.0)
Avoiding	0 (0.0)	13 (13.0)	66 (66.0)	20 (20.0)	1 (1.0)
Sensitivity	0 (0.0)	20 (20.0)	60 (60.0)	19 (19.0)	1 (1.0)
Registration	0 (0.0)	9 (9.0)	60 (60.0)	26 (26.0)	5 (5.0)
General Processing	0 (0.0)	1 (1.0)	44 (44.0)	13 (13.0)	42 (42.0)
Auditory Processing	0 (0.0)	0 (0.0)	47 (47.0)	34 (34.0)	19 (19.0)
Visual Processing	0 (0.0)	56 (56.0)	44 (44.0)	0 (0.0)	0 (0.0)
Touch Processing	0 (0.0)	0 (0.0)	27 (27.0)	21 (21.0)	52 (52.0)
Movement Processing	18 (18.0)	14 (14.0)	50 (50.0)	15 (15.0)	3 (3.0)
Oral Processing	0 (0.0)	0 (0.0)	62 (62.0)	16 (16.0)	22 (22.0)
Behavioral Responses	0 (0.0)	0 (0.0)	44 (44.0)	8 (8.0)	48 (48.0)

Categories are age-referenced ITSP-2 classifications. Percentages sum to 100% within each dimension.

**Table 5:** Multiple linear regression model for Infant Colic Scale scores.

Predictor	B	SE	β	t	*p*	95% CI for B
Constant	74.855	4.345	—	17.227	<0.001	66.23 to 83.48
Iowa Infant Feeding Attitude Scale	−0.236	0.060	−0.291	−3.947	<0.001	−0.355 to −0.117
Sensation Avoiding	−0.170	0.072	−0.146	−2.341	0.021	−0.313 to −0.027
General Processing	0.299	0.041	0.567	7.232	<0.001	0.218 to 0.380
Auditory Processing	−0.172	0.077	−0.125	−2.243	0.027	−0.325 to −0.019

B = unstandardized coefficient; SE = standard error; β = standardized coefficient; CI = confidence interval. Model: R^2^ = 0.728; adjusted R^2^ = 0.716; F(4, 95) = 63.498, *p* < 0.001.

**Table 6:** Multiple linear regression model for Iowa Infant Feeding Attitude Scale scores.

Predictor	B	SE	β	t	*p*	95% CI
Constant	97.010	6.559	—	14.791	<0.001	83.993 to 110.027
Infant Colic Scale score	−0.526	0.125	−0.427	−4.222	<0.001	−0.774 to −0.279
Low Registration	−0.449	0.121	−0.376	−3.720	<0.001	−0.688 to −0.209

B = unstandardized coefficient; SE = standard error; β = standardized coefficient; CI = confidence interval. Model: R^2^ = 0.564; adjusted R^2^ = 0.555; F(2, 97) = 62.765, *p* < 0.001.

## Data Availability

The corresponding author will provide the data in detail upon request.

## Abbreviations

The following abbreviations are used in this manuscript:

CI	Confidence interval
ICS	Infant Colic Scale
IIFAS	Iowa Infant Feeding Attitude Scale
ITSP-2	Infant/Toddler Sensory Profile-2
SD	Standard deviation
SE	Standard error
VIF	Variance inflation factor
